# Association between Donor Age and Osteogenic Potential of Human Adipose Stem Cells in Bone Tissue Engineering

**DOI:** 10.3390/cimb46020092

**Published:** 2024-02-06

**Authors:** Md Abdus Sattar, Lara F. Lingens, Vincent G. J. Guillaume, Rebekka Goetzl, Justus P. Beier, Tim Ruhl

**Affiliations:** Department of Plastic Surgery, Hand Surgery—Burn Center, University Hospital, Rheinisch-Westfälische Technische Hochschule Aachen, 52074 Aachen, Germany; msattar@ukaachen.de (M.A.S.); llingens@ukaachen.de (L.F.L.); vguillaume@ukaachen.de (V.G.J.G.); rgoetzl@ukaachen.de (R.G.); jbeier@ukaachen.de (J.P.B.)

**Keywords:** ASC, osteogenic potential, osteoblast, aging, regenerative medicine, tissue engineering

## Abstract

Adipose stem cells (ASCs) have multilineage differentiation capacity and hold great potential for regenerative medicine. Compared to bone marrow-derived mesenchymal stem cells (bmMSCs), ASCs are easier to isolate from abundant sources with significantly higher yields. It is generally accepted that bmMSCs show age-related changes in their proliferation and differentiation potentials, whereas this aspect is still controversial in the case of ASCs. In this review, we evaluated the existing data on the effect of donor age on the osteogenic potential of human ASCs. Overall, a poor agreement has been achieved because of inconsistent findings in the previous studies. Finally, we attempted to delineate the possible reasons behind the lack of agreements reported in the literature. ASCs represent a heterogeneous cell population, and the osteogenic potential of ASCs can be influenced by donor-related factors such as age, but also gender, lifestyle, and the underlying health and metabolic state of donors. Furthermore, future studies should consider experimental factors in in vitro conditions, including passaging, cryopreservation, culture conditions, variations in differentiation protocols, and readout methods.

## 1. Introduction

**Figure 1:**
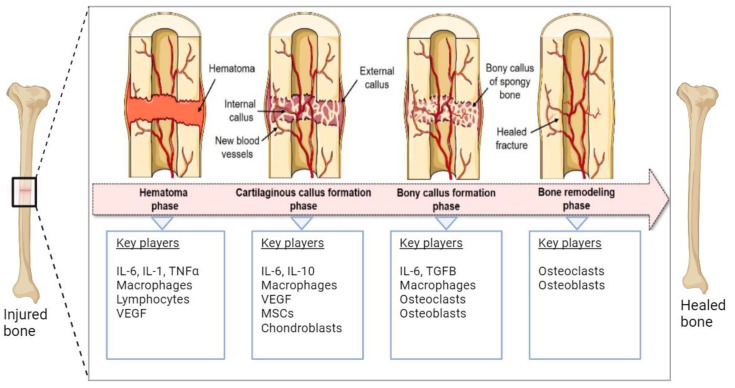
Schematic representation of the natural bone repair process. Immune cells associated with hematoma are formed in the first phase. In the second phase, MSCs are recruited and differentiated into chondroblasts and osteoblasts that help the formation of soft cartilage callus. VEGFs promote vascularization in this phase. In the third phase, soft cartilage is replaced by hard bone facilitated by osteoblasts, osteoclasts, chondroclasts, and chondroblasts. The final step involves the continuous remodeling of newly formed bone. The figure was modified from Pfeiffenberger et al. [1], licensed under a Creative Common Attribution (CC BY) 4.0 Generic License, https://creativecommons.org/licenses/by/4.0/) (accessed on 24 November 2023). Created with BioRender.com (accessed on 24 November 2023).

Human bone tissue can regenerate spontaneously after fracture or injury through a bone remodeling process consisting of four phases (Figure 1). Within the first five days after injury, the fracture hematoma is formed accompanied by local inflammation, resulting in the activation and migration of immune cells, including macrophages, monocytes, and lymphocytes [1,2]. Next, the regenerating cells of the connective tissue, including mesenchymal stem cells (MSCs), are recruited to the site of injury, and the fibrocartilaginous fracture callus is formed by the rapid proliferation and differentiation of MSCs into chondroblasts, osteoblasts, and fibroblasts. The differentiation of MSCs is induced by several mediators of cell differentiation such as bone morphogenic proteins (BMPs). The expression and localization of BMPs (e.g., BMP-2 and BMP-4) during early fracture healing have been reported in the literature [3,4]. The vascular endothelial growth factors (VEGFs) secreted by immune cells promote angiogenesis through the formation of new blood vessels. The third phase involves the endochondral ossification of the cartilage where soft cartilage is replaced by a hard bone callus. VEGF-mediated vascularization continues deeper into the callus and facilitates further migration of MSCs and the supply of oxygen and nutrition. Finally, the newly formed bone callus is continuously remodeled by the resorption of osteoclasts and the formation of osteoblasts [1,5,6].

This spontaneous bone healing is a multilateral process that is regulated by various intrinsic and extrinsic factors and can be disrupted by various causes at various time points, resulting in a failure to heal successfully [7]. Modern surgical techniques augment natural as well as impaired healing by grafting autologous, allogenic, or prosthetic materials to the recipient site [8]. However, the transfer of autologous material is limited by the availability of appropriate tissue, and it holds the risk of donor-site morbidity, unpredictable graft absorption, infections, and structural failure.

To address these issues, bone repair through osteogenic tissue engineering could combine suitable progenitor cells with appropriate scaffolds and growth factors [2]. Autologous stem cell transplantation includes cell isolation and the subsequent in vitro expansion, eventually followed by the transplantation into the defect site. However, this strategy is challenged by the availability of suitable stem cell populations with sufficient intrinsic potential for osteogenic differentiation. Admittedly, age-related changes in stem cell characteristics are an important criterion that should be taken into account when considering autologous stem cell therapy for bone tissue regeneration and/or artificial bone tissue engineering. Although the clinical applicability of MSCs spans all age groups, elderly people are the primary beneficiaries of stem cell-based regenerative medicine because degenerative bone diseases, and delayed or impaired fracture healing are more prevalent in the elderly population. In addition, the risk for bone deformation and fractures per se increases with age, while it is associated with a decreased ability for tissue regeneration and repair. Age-associated microenvironmental changes, such as metabolic alteration, hormonal disturbance, and immunological disorders, might also affect the stem cell niche and, thus, impair the regenerative potential of the MSCs [9]. Moreover, aging induces profound changes in various molecular, genetic, and epigenetic processes, resulting in alterations to the proliferation and differentiation potential of MSCs. Ultimately, this leads to disrupted tissue homeostasis and impaired repair abilities [10]. Therefore, it is not only important to find a suitable autologous cell population with the potential to regenerate bone tissue but also crucial to choose an MSC type that is less affected by age. The most promising candidates are adipose stem cells (ASCs) and MSCs derived from bone marrow (bmMSCs), members of the MSC family, which share unique features for osteogenic differentiation [11,12].

bmMSCs have multilineage differentiation potential, and their use in regenerative medicine is not restricted by ethical issues. However, as discussed in previous reviews [13,14,15,16,17], several disadvantages restrict the use of bmMSCs in bone tissue engineering. For example, bmMSC isolation procedures can be associated with donor-site morbidities such as pain, infection, hematomas, seromas, nerve injuries, arterial injuries, and fractures [18]. In addition, aging negatively impacts bmMSC harvests because the cell yield declines with age [19]. Moreover, a negative age effect is observed in both the proliferation as well as osteogenic differentiation potential of bmMSCs [19,20,21,22]. Additionally, bmMSCs isolated from elderly donors exhibit increased senescence properties and ROS-induced oxidative damage [20,21,22]. It has been postulated that the bmMSCs from elderly donors favor adipogenic differentiation instead of osteogenic differentiation, a process termed the “adipogenic switch” [13,14,17].

Compared to bmMSCs, ASCs are abundantly available, can be isolated through a minimally invasive liposuction procedure, and yield a higher number of cells [23]. Meyer et al. obtained nearly 2–3 million cells with stem cell properties from a mere 10 mL sample of lipoaspirate [24]. ASCs hold the capacity to differentiate into adipocytes, chondrocytes, osteoblasts, skeletal muscle cells, and tenocytes, in vitro [25]. The regenerative potential of ASCs and ASC-derived secretomes have been substantiated in numerous studies, and when combined with 3D scaffolds and microfluidic systems, ASCs support bone as well as soft tissue repair and regenerative processes [25]. For example, a case report involving 52 males diagnosed with stage I avascular necrosis (AVN) of the femoral head and treated with core decompression and injection of ASCs from abdominal liposuction reported the healing of the bone lesion after three months [26].

A clear and widely accepted definition of ASCs is yet to be determined, particularly concerning the unique expression of cell surface markers [27]. In 2006, the International Society for Cell and Gene Therapy (ISCT) proposed three main criteria of MSCs, namely their ability to adhere to the plastic surface; being positive for CD73, CD90, CD105, and negative for CD45, CD34, CD14, CD19, and HLA-DR; and their ability to differentiate into adipocytes, osteoblasts, and chondrocytes. In 2013, the International Federation for Adipose Therapeutics and Science (IFATS) and ISCT defined phenotypic markers specific to the ASC population [28]. According to the joint statement, the stromal vascular fraction (SVF) obtained by the collagenase digestion of adipose tissue should be characterized by CD31^−^, CD45^−^, CD235a^−^, and CD34^+^. The cultured ASCs should retain the markers of MSCs (being positive for CD44, CD73, CD90, and CD105) and can be distinguished from bmMSCs by CD36^+^ and CD106^−^.

The mechanism through which ASCs promote bone fracture healing is not clearly understood. It was suggested that the bone repair process by ASCs is primarily mediated by their paracrine activity, i.e., by the release of soluble factors such as growth factors, cytokines, chemokines, and extracellular vesicles. Among others, the ASC secretome includes immunomodulatory cytokines (e.g., interleukins and TNFa), and proangiogenic factors (e.g., VEGF and HGF). As shown in Figure 1, multiple factors contribute to fracture healing, including immune cells, growth factors, stem cells, chondroblasts, osteoblasts, and osteoclasts. The ASC secretome supports bone healing by recruiting immune cells, supporting osteogenic differentiation of progenitor cells, and promoting the generation of new blood vessels (angiogenesis) at the site of injury [2]. Localized differentiation mediators such as BMP-4 can induce the differentiation of ASCs into osteoblasts [29], which may further accelerate the bone repair process. Although the ASC-mediated acceleration of the bone repair process can be achieved by both differentiated and undifferentiated ASCs [30], it was reported that osteogenically induced ASCs perform better in fracture healing than uninduced ASCs [31].

Despite the great potential of ASCs in artificial bone tissue engineering, age-related changes in ASC functions remain elusive. Understanding the age-associated changes in ASC osteogenesis is of high importance when determining the optimal therapeutical applications of ASCs.

Researchers explored the effect of aging on the regenerative potential of ASCs in many studies. However, inconsistent results have been reported in the literature. Dufrane (2017) reviewed the impact of age on ASC isolation, the risk of oncogenicity, and bone tissue engineering and concluded that adipose cell properties are not dependent on donor age [32]. A systematic review from 2017 analyzed the results from 41 in vitro, in vivo, and clinical studies and found a decreased proliferation and differentiation potential of ASCs with increasing age [33]. In the present review, we aimed to update the current understanding of the impact of age on the osteogenic potential of human ASCs. We excluded studies that isolated ASCs from non-human sources because studying human aging in short-lived animal models remains controversial [34]. Furthermore, ASCs derived from non-human species are not suitable for transplantation into humans due to immunological disparities. To the best of our knowledge, this is the first review that focuses exclusively on the effect of age on the osteogenic potential of ASCs derived from humans.

**Figure 2:**
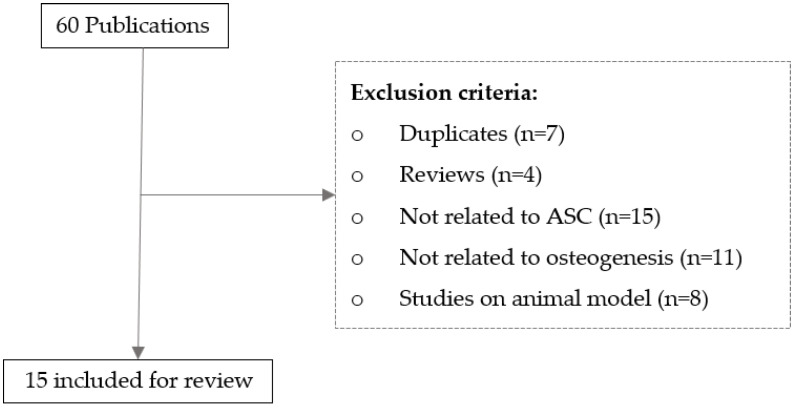
Flowchart demonstrating the selection process in this review.

A literature search was performed using Google Scholar and PubMed databases, and a total of 65 papers published between 2005 and 2021 were identified. Then, 50 papers were excluded, and 15 papers based on primary research involving human (h)ASCs met the criteria and were included in the final review (Figure 2). Among the 15 reviewed studies, 8 studies recruited both males and females, 6 studies included females only, and the gender of the donors was unspecified in 1 study. As summarized in Table 1 and reviewed in the following section, little consensus has been reached in the literature regarding the effect of donor age on the osteogenic potential of hASCs.

## 2. Review of Previous Studies

Among the 15 original studies included in this review, 7 studies reported no significant influence of age on the osteogenic differentiation capacity of hASCs. For example, Chen et al. evaluated the effect of age on the osteogenic potential of hASCs from 14 young patients with hip fracture (36.4 ± 11.8 years) and 8 elderly patients (71.4 ± 3.6 years) with osteoporosis. The results from their study indicate that age is not an influential factor in terms of matrix mineralization and the calcification of hASCs. However, the mRNA expression of *osteocalcin* and *ALP* genes decreased in the hASCs of elderly donors [37]. Wu et al. investigated the effect of age on the osteogenic potential of hASCs isolated from infant (<1 year), adult (20–54 years), and elderly (>55 years) donors. Although *RUNX2* and *osteocalcin* mRNA expression and matrix mineralization were higher in infant ASCs, these parameters were overall comparable to those from adult and elderly donors [38]. A complex relationship between donor age and the osteogenic potential of hASCs from female donors was revealed in a study by Zhu et al. Age-associated decline in hASC osteogenesis was not significant in the study population (20–58 years). However, a significant decline in osteogenesis, in terms of decreased matrix calcification in the von Kossa staining, was observed when female donors entered their 40s, which suggests that these changes may be associated with estrogen loss during the transition of women from pre- to perimenopause [35]. To overcome the high interindividual variability between various donors, Bodle et al. generated ASC “superlots”, i.e., pooled donor cell populations derived from four to five age-clustered premenopausal (24–36 years), perimenopausal (48–55 years), and postmenopausal (60–81 years) female donors. With this superlot approach, the authors revealed that despite high donor-to-donor variability, young hASCs are primed to the adipogenic lineage, whereas old hASCs (60–81 years) preferentially differentiate osteogenically [40]. A report from Kawagishi-Hotta and colleagues included hASCs from a large number of donors (n = 260) aged 5–97 years old. The authors concluded that age negatively impacts adipogenic differentiation but not chondrogenic and osteogenic differentiation, measured by Oil Red O staining, sulfated glycosaminoglycan content, and Alizarin Red S staining, respectively. The principle component analysis (PCA) for ASC characteristics revealed that the proliferation and multilineage differentiation varied in each individual, particularly in females at an age of >60 years. Another study investigated the differentiation potential of hASCs derived from nine young (<36 years) and nine elderly (>54 years) donors. After osteogenic induction for up to four weeks, no significant differences were observed between hASCs of young and elderly donors in terms of matrix mineralization, evaluated by the OsteoImage™ Mineralization Assay, and the expression levels of osteogenic genes (*RUNX2* and *CEBPA*), measured by quantitative PCR [47].

In contrast, eight studies found a diminishing effect of donor age on the osteogenic function of hASCs. For instance, Alt et al. presented a correlation between age-related changes in the quality of stem cells and differentiation capabilities using hASCs from young (<20), middle-aged (30–40), and elderly (>50) healthy donors. They observed an age-dependent downregulation of miRNAs (*mir-27B* and *let-7G*), which regulate the cell cycle, apoptosis, and inhibition of the multilineage potential of hASCs [10]. Similarly, Choudhery et al. studied the influence of age on the in vitro differentiation of hASCs from young (<30), adult (35–50 years), and aged (>60 years) individuals. In this study, the cells from aged donors displayed higher cellular senescence (confirmed by increased SA-β-gal staining), which correlated with a lowered level of cell mineralization in von Kossa staining compared to their young counterparts [39]. The association between donor age and the differentiation potential of human orbital adipose-derived stem cells (OASCs) was investigated by Ye et al. [41]. OASCs were isolated from the lower eyelid of young (20–38 years) and adult (50–67 years) female individuals who underwent routine blepharoplasty. OASCs from older donors displayed increased expression levels of senescence-related genes (*p21* and *p53*) as well as decreased calcium deposition detected in the Alizarin Red assay [41]. Similarly, in another study, human eyelid ASCs showed decreased Alizarin Red staining for matrix calcification and a decreased expression of the osteoblastic gene *OPN* [46].

Marędziak and colleagues isolated hASCs from the subcutaneous fat of 28 healthy donors divided into four age groups: >20 years, >50 years, >60 years, and >70 years. They confirmed that the age group classified as younger (20–49 years) displayed a higher level of matrix calcification in Alizarin Red assay and an increased expression of osteogenic factors (*osteocalcin*, *BMP-2*, and *osteopontin*) in RT-PCR and ELISA, compared to age group >50 years [42,49]. A similar observation was reported by Liu et al. in the following year on hASCs from adipose tissue of children (6–12 years), adults (22–27 years), and elderly individuals (60–73 years) [43]. They observed an age-associated increased cellular senescence manifested by an increase in SA-β-gal-positive cells, as well as a decline in osteogenic potential marked by the downregulation of osteogenic genes (*RUNX2*, *BMP-2*, *osteocalcin*, and *osteopontin*) in RT-PCR and decreased matrix calcification in Alizarin Red staining in hASCs from elderly donors [43].

Since the regenerative potential of hASCs to support local tissue repair is predominantly attributed to their paracrine activity, a recent study illustrated that the age-altered secretory patterns of hASCs lead to the reduced release of vascular endothelial growth factor (VEGF), hepatocyte growth factor (HGF), and stromal cell-derived factor 1-α. In addition, hASCs from elderly donors (>70 years) rarely differentiated into osteoblasts compared to hASCs derived from younger (<30 years) donors, as hASCs from the younger individuals revealed significantly higher calcium deposition in the Alizarin Red assay. Although the secretion of BMP-2 protein was similar among both groups, the expression of its receptor (BMPR1A) was lower in the elderly group. Thus, the author postulated that elderly hASCs might exhibit a weaker response to the BMP-2 protein due to the reduced expression of its receptor [48]. Together, emerging evidence suggests that age impairs the osteogenic potentials of hASCs.

## 3. Discussion

As summarized in Table 1, there is conflicting evidence regarding the effect of age on the osteogenic differentiation potential of hASCs. Some studies found no significant effect of donor age on the osteogenic potential of the cells, while other studies depicted a deteriorating effect. This inconsistency could be dependent on several limitations in the included studies. For example, the sample size could have been too small to generate statistically significant data. In just one study, hASCs were isolated from a large number of donors (n = 260 donors, aged 5–97 years) and demonstrated an age-dependent adverse effect on adipogenesis but not on osteogenesis or chondrogenesis [44]. As MSCs usually maintain a differentiation balance—if one differentiation lineage is favored, the other one is inhibited—the findings of the previously mentioned study partially support this paradigm [17].

Since there is no standard for age clustering, researchers grouped individuals in various ways. Often, the selected age range was not large enough to make a valid comparison. In other words, in many earlier observations, the age difference between young and elderly donors was small, which might have concealed a true age effect. Studies on hASCs isolated from donors with a narrower age range found no significant impact of age. For example, in a study by Horinouchi et al., the osteogenic potential of young individuals (>34 years) and adults (<54 years) remained unaffected by age in terms of bone mineralization and the expression levels of osteogenic genes (*RUNX2* and *CEBPA*) [47]. In contrast, Park reported age-related alterations in bone mineralization and *BMP-2* gene expression in hASCs from two groups of donors with a wider age range (<30 years vs. >70 years) [48]. Similarly, two studies included infants (<1 year) or children (6–12 years) and demonstrated that hASCs from infants and children have higher osteogenic potentials in comparison to hASCs from elderly people (>55 years) [38,43].

Notably, even though this review focuses on hASCs, some in vivo mouse models also reported a similar age-dependent effect when comparing ASCs isolated from mice with large age differences. For example, age-related alterations in ASC proliferation and differentiation were reported by Li and Doshida et al. in the same species but with distinct age groups, i.e., 1-month-old vs. 20-month-old, and 6-month-old vs. 29-month-old, respectively [50,51,52]. However, Shi et al. observed no significant effect of age on the differentiation potential of ASCs from 6-days-old and 60-days-old mice.

Thus, age-related changes in the osteogenic potential of hASCs might be visible when hASCs from very young and very old donors with distinct age differences are compared. As younger individuals are less likely to undergo surgery, hASCs from young donors are significantly harder to recruit for experimental studies. This might be the reason why previous studies did not investigate hASCs from distinct age groups. For the same reason, hASCs were obtained predominantly from females rather than males because females are more likely to undergo plastic surgery [53]. Interestingly, out of the six studies that recruited female donors only, three studies considered age range related to the perimenopause (40–50 years) and revealed that hASCs obtained from female individuals in their early 40s exhibited increased lipid accumulation and decreased potential to differentiate into osteogenic lineage compared to hASCs from younger (<30 years) and older (>55 years) women [35,40,47,54]. These authors postulate that menopause-related changes in estrogen levels could explain this transient effect of age on hASC function. It is well known that a declined estrogen level is associated with low-grade inflammation that triggers fat accumulation and activates osteoclasts to degrade bone tissue [55]. In conclusion, gender and menopausal status should be considered when grouping donors based on age, and future studies should further explore the effects of hormonal changes and osteoporosis on ASC properties.

Some authors reported high intragroup variability in hASC characterization and differentiation, which could conceal age-dependent effects This apparent high donor-to-donor variability could be attributed to other demographic and lifestyle factors, e.g., general health status, medical and disease history, body mass index, or epigenetic patterns related to the environment; donor habits may also influence experimental outcomes, as reviewed by Prieto González in 2019 [56]. These donor characteristics have been disregarded in the literature, and in many cases, BMI was used as the sole parameter to describe the obesity status of individuals [10,35,36,37,41,43,44,47]. Increased BMI as a marker of obesity is associated with a decreased osteogenic potential of hASCs [57]. However, the role of BMI in identifying people with obesity is controversial as it cannot distinguish fat from muscle or bone mass. Therefore, a more useful indicator of obesity should be used when defining non-obese donors of ASCs.

Studies evaluating the effect of age on bone tissue engineering used hASCs from diverse anatomical sites, including the abdomen, the epididymis, and the eyelid. Surgical methods of fat harvesting also varied between the presented studies. Differences in the anatomical origin of adipose tissue and surgical procedures may be the underlying confounder since hASCs from different donor sites and methods of extraction exhibit distinct biological properties [58]. For instance, Requicha et al. assessed the expression profile of osteogenic genes (*COLIA1*, *RUNX2*, and *Osteocalcin*) of hASCs from the canine subcutaneous and omental origin by RT-PCR analysis. While *RUNX2* expression did not differ between the two fat depots, *COLIA1* had significantly higher expression in subcutaneous hASCs, whereas *osteocalcin* displayed an inverse expression pattern [59].

**Figure 3:**
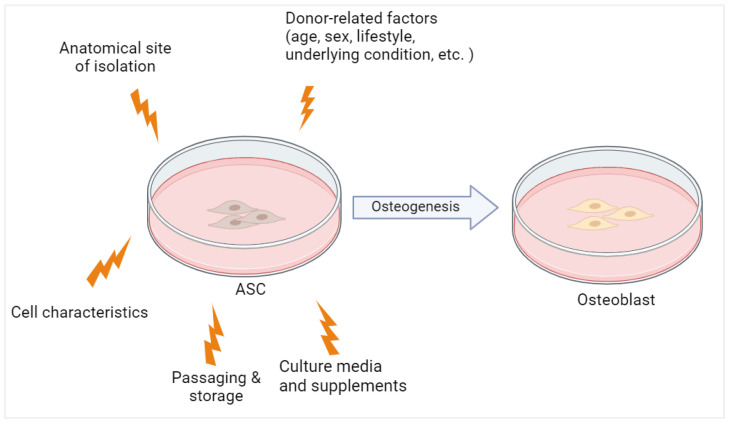
The osteogenic potential of ASC is influenced by both donor-related and experimental factors. Created with BioRender.com (accessed on 24 November 2023).

Apart from donor-related factors, the proliferation as well as differentiation potential of hASCs are also influenced by long-term passage, cryopreservation, and culture conditions (Figure 3), as these parameters varied in previous reports [56,60]. Often, osteogenic induction was performed on cryopreserved cells, after passages 1 to 5 by using osteogenic induction media with different compositions. Furthermore, previous studies selected different endpoints as the marker of osteogenic differentiation with various readout methods. Growth factors and serum supplementation also greatly differ between laboratories. These experimental variations may influence hASC stemness, proliferation, and differentiation [56].

As mentioned before, an accurate definition of ASCs remains an open issue. Among the 15 included studies, 8 studies characterized ASCs based on 2006 ISCT criteria of antigen expression profile for MSCs (Table 2), while 7 studies characterized ASCs by plastic adherence property and tri-lineage differentiation capacity. These characteristics are common for all MSCs and therefore a distinction between ASCs and other MSCs was not apparent. None of the studies included specific markers of ASCs in SVF (CD31^−^, CD45^−^, CD235a^−^, and CD34^+^) and in culture (CD36^+^ and CD106^−^) jointly proposed by the IFATS and ISCT in 2013. Thus, a lack of a uniform method of characterization of ASCs could contribute to conflicting results in previous studies.

## 4. Conclusions

The effect of age on the osteogenic differentiation potential of hASCs has been highly debated in the literature, and hitherto, poor agreement has been achieved in previous studies. Factors that might contribute to a disagreement in previous research include experimental variables such as small sample size, the lack of standard age grouping, differences in protocols for osteogenic differentiation and readout methods, as well as donor-related factors, for instance, hormonal status, underlying disease conditions, and metabolic status of hASC donors. Apart from the effect of age, future studies should also consider the intrinsic and extrinsic factors that may influence the osteogenic potential of hASCs. Attempts should be made to minimize heterogeneity through the purification of ASCs based on unique cell surface markers.

## Figures and Tables

**Table 1:** Summary of studies included in this review.

Year	Aim	Study Design	Methods	Results	Conclusions	Other Variables	Ref.
2009	Effect of donor age on differentiation potential of ASCs.	ASCs from females aged 20–58 years; N = 27, n = 9, 7, 11.	ALP activity after 7 days Ca^2+^ deposition by von Kossa after 4 weeks	↓ ALP Activity with age ↓ Ca^2+^ deposition with age	Osteogenic differentiation decreases with age	SC: female, non-obese AO: u.s (liposuction) Passage: 1 OI day: up to 28 days	[35]
2009	In vitro differentiation potential of ASCs from young and elderly females.	Young (<35) and older (>45) females N = 26.	ALP activity Assay after 14 days Ca^2+^ deposition by Alizarin Red assay after 21 days	↓ ALP activity with age No significant difference in Ca^2+^ deposition	Donor age mildly affects the potential of ASCs for osteogenic differentiation in vitro	SC: non-obese female, BMI < 30 AO: subcutaneous (lipo-asp.) Passage: 1 OI day: 21 days	[36]
2012	Age-associated changes in molecular characteristics of ASCs.	ASCs from healthy young (<20), middle-aged (30–40), and elderly (>50) donors. N = 40; n = 15, 17, 8.	Ca^2+^ deposition by Alizarin Red S ALP activity assay Osteogenic gene (*BMP-6*, *COL2A*, *COL10A*) expression by RT-PCR	↓ Ca^2+^ deposition ↓ ALP activity ↓ Expression of osteogenic genes	Aging processes significantly attenuate the osteogenic differentiation potential of ASCs	SC: healthy male/female, BMI < 29 AO: abdominal Passage: u.s OI day: u.s	[10]
2012	Effect of age on ASCs and bmMSCs from elderly patients with osteoporosis.	ASCs from young (<36) and elderly (>67) individuals N = 22, n = 14, 8.	Ca^2+^ deposition by Alizarin Red S	No significant difference in Ca^2+^ deposition	The osteogenic differentiation of ASCs is not impacted by age	SC: male/female, osteoporotic, BMI < 26 AO: gluteal subcutaneous Passage: 5 OI day: up to 14 days	[37]
			Osteogenic genes (*OCA*, *BMP2*, *RUNX2*, and *ALP*) by RT-PCR	No significant difference in gene expression.			
2012	Effect of aging on senescence, osteogenic factors, and osteogenesis of ASCs.	ASCs from infants (<1), adults (20–54), and elderly individuals (>55), N = 13; n = 4, 6, 3.	Senescence (TL) by RT-PCR *RUNX2*, *osteocalcin* by RT-PCR ALP activity assay Ca^2+^ deposition by Alizarin Red S	↑ Senescence with age ↓ Osteogenic gene expression compared to infant ↓ ALP activity and Ca^2+^ deposition compared to infant.	Biological properties are conserved during the adult to the elderly period (but not compared to infants)	SC: male/female. AO: abdominal (liposuction) Passage: 1 OI day: up to 21 days	[38]
2014	Impact of age on the quality of human adipose tissue-derived MSCs.	ASCs of young (<30), adult (35–50), and elderly (>60) donors, N = 29; n = 10, 8, 11.	Ca^2+^ deposition by von Kossa staining Senescence by β-galactosidase Staining Osteogenic genes (*osteocalcin* and *ALP*) by RT-PCR	↓ Ca^2+^ deposition with age ↑ Senescence with age ↓ Expression of osteocalcin and ALP with age.	Age negatively impacts stem cell osteogenic differentiation	SC: male/female AO: (liposuction) Passage: 2–3 OI day: 21 days	[39]
2014	Effect of age on osteogenesis of female ASCs: superlot approach.	ASCs from female patients (24–81), superlot biobanking. N = 14; n = 5, 4, 5.	Ca^2+^ deposition by Alizarin Red S	↑ Ca^2+^ deposition with older (postmenopausal) female.	Existence of a high degree of donor-to-donor variations which is independent of age	SC: female AO: u.s Passage: 1 OI day: 14 days	[40]
2016	Effects of donor age on the biological properties of human OASC.	OASCs from young (20–38, normal) and old donors (50–67, fat pad in lower eyelid) N = 20; n = 10, 10.	Ca^2+^ deposition by Alizarin Red S Ca^2+^ deposition by Von Kossa staining	↓ Ca^2+^ deposition with age	The benefit of autologous OASCs from elderly patients for osteogenic therapeutic purposes may be limited	SC: female, non-obese AO: lower eyelid fat pad Passage: 3 OI day: 14 days	[41]
2016	Effect of age on the osteogenic potential of ASCs.	ASCs from different age groups: >20 y, >50 y, >60, >70 N = 32; n = 8.	Ca^2+^ deposition by Alizarin Red ALP activity assay Osteogenic markers (*OPN*, *Col-I*, *OCL*, and *BMP-2*) by PCR	↓ Ca^2+^ deposition with age No significant difference in ALP activity ↓ Expression of osteogenic markers with age	Age negatively influences the osteogenic potential of ASCs	SC: healthy male/female AO: subcutaneous Passage: 1 OI day: 21 days	[42]
2017	Systematical analysis of the effects of age on the quantity and quality of ASCs.	ASCs were isolated from children (6–12), young individuals (22–27), adults (60–73), and the elderly, N = 24; n = 10, 8, 6.	Cellular senescence assay Ca^2+^ deposition by Alizarin Red S Osteogenic genes (*RUNX2*, *ALP*, *OCN*, and *OPN*) by RT-PCR	ASCs from elderly donors exhibit senescent properties. ASCs from aged patients exhibit impaired osteogenic potential	While ASCs from different age populations are phenotypically similar, they present major differences at the functional level	SC: male/female BMI < 22 AO: chest subcutaneous Passage: 3 OI day: up to 21 days	[43]
2017	Effect of donor age on differentiation potential of ASCs.	ASCs of 260 donors (ages 5–97 years) N = 260.	Ca^2+^ deposition by Alizarin Red S	The osteogenic potential (marked by Ca^2+^ deposition) of ASCs does not correlate with donor age	The chondrogenic and osteogenic potential of ASCs were not affected by age	SC: male/female, median BMI = 22.7 AO: subcutaneous Passage: 5 OI day: 21 days	[44]
2017	Cell–substrate impedance spectroscopy (ECIS) to track complex bioimpedance pattern of ASC osteogenesis.	ASC superlot from young (24–36), middle-aged (48–55), and elderly (60–81) adults.	ECIS measurement throughout the osteogenic differentiation phases	ASCs from younger donors require a longer time to differentiate than ASCs from older donors.	Donor age may temporally control the onset of osteogenesis	SC: female AO: u.s (liposuction) Passage: u.s CS: u.s OI day: u.s	[45]
2018	Effect of donor age on the regenerative potential of HEASCs.	HEASCs from <20 y, >20 y, <45 y, >55 y N = 13; n = 4, 5, 4.	Ca^2+^ deposition by Alizarin Red S *RUNX2* by RT-PCR	↓ Ca^2+^ deposition with age No difference in gene expression.	Donor age has a negative influence on the osteogenic differentiation of HEASCs	SC: healthy donor AO: eyelid Passage: 5 OI day: 21 days	[46]
2020	Differentiation potential of ASCs isolated from the lipoaspirates of elderly and young donors.	ASCs from young (<34) and old (>54) female donors, N = 18; n = 9, 9.	Cell mineralization assay *RUNX2* by RT-PCR	No significant difference No significant difference	Age does not significantly impact the osteogenesis of ASCs	SC: female, BMI < 30; AO: u.s (liposuction) Passage: 4–7 OI day: up to 28 days	[47]
2021	Association between age and ASC differentiation potential.	ASCs from young (<30) and elderly (>70), N = 8.	Ca^2+^ deposition by Alizarin Red S BMP-2 by ELISA BMP-2 receptor by WB	↓ Ca^2+^ deposition with age No significant difference ↓ BMP-2 with age	Age may affect the cellular function and differentiation of ASCs	SC: healthy male/female, AO: u.s Passage: 3–5 OI day: 20 days	[48]

Abbreviations: TL = telomere length; GF = growth factor, ns = not significant, rhBMP-2 = recombinant human bone morphogenic protein 2; HEASC = Human eyelid adipose stem cell, DM = diabetic mellitus; ECM = extracellular matrix, ECIS = cell–substrate impedance spectroscopy, OASC = orbital adipose-derived stem cell, GF = growth factor, OCA = osteocalcin, BMP = bone morphogenic protein, ALP = alkaline phosphatase, N = total number of participants/animal, n = sample size (number in each group), SC = sample characteristics, AO = anatomical origin, CS = centrifugal speed, OI = osteogenic induction, u.s = unspecified, Ns = not significant, ↑ = increasing/upregulated, ↓ = decreasing/downregulated.

**Table 2:** ASC characterization methods described in the included studies.

Method	Positive Markers	Negative Markers	References
Flow Cytometry	CD44, CD90, CD105, CD146	CD3, CD4, CD11b, CD34, CD45	[10]
Flow Cytometry	CD44, CD73, CD90,CD105	-	[38]
Flow Cytometry	CD44, CD73, CD90, CD105	CD3, CD14, CD19, CD34, CD45	[39]
Flow Cytometry	CD73, CD90, CD105	CD14, CD19, CD34, CD45	[41]
Flow Cytometry	CD44, CD73, CD90, CD105	CD34, CD45	[42]
Flow Cytometry	CD44, CD73, CD90, CD105	CD34, CD11b, CD19, CD45, HLA-DR	[43]
RT-qPCR	CD44, CD73, CD90, CD105, CD271, NANOG	-	[44]
Flow Cytometry	CD44, CD73, CD90, CD105	CD31, CD34, CD45	[46]
